# Prevention of mother-to-child transmission of HIV: Postpartum adherence to Option B+ until 18 months in Western Uganda

**DOI:** 10.1371/journal.pone.0179448

**Published:** 2017-06-29

**Authors:** Sarah Decker, Eva Rempis, Alexandra Schnack, Vera Braun, John Rubaihayo, Priscilla Busingye, Nazarius Mbona Tumwesigye, Gundel Harms, Stefanie Theuring

**Affiliations:** 1 Institute of Tropical Medicine and International Health, Charité- Universitätsmedizin, Berlin, Germany; 2 Public Health Department, Mountains of the Moon University, Fort Portal, Uganda; 3 Holy Family Virika Hospital, Fort Portal, Uganda; 4 School of Public Health, College of Health Sciences, Makerere University, Kampala, Uganda; National and Kapodistrian University of Athens, GREECE

## Abstract

Since 2012, the WHO recommends Option B+ for the prevention of mother-to-child transmission of HIV. This approach entails the initiation of lifelong antiretroviral therapy in all HIV-positive pregnant women, also implying protection during breastfeeding for 12 months or longer. Research on long-term adherence to Option B+ throughout breastfeeding is scarce to date. Therefore, we conducted a prospective observational cohort study in Fort Portal, Western Uganda, to assess adherence to Option B+ until 18 months postpartum. In 2013, we recruited 67 HIV-positive, Option B+ enrolled women six weeks after giving birth and scheduled them for follow-up study visits after six, twelve and 18 months. Two adherence measures, self-reported drug intake and amount of drug refill visits, were combined to define adherence, and were assessed together with feeding information at all study visits. At six months postpartum, 51% of the enrolled women were considered to be adherent. Until twelve and 18 months postpartum, adherence for the respective follow-up interval decreased to 19% and 20.5% respectively. No woman was completely adherent until 18 months. At the same time, 76.5% of the women breastfed for ≥12 months. Drug adherence was associated with younger age (p<0.01), lower travel costs (p = 0.02), and lower number of previous deliveries (p = 0.04). Long-term adherence to Option B+ seems to be challenging. Considering that in our cohort, prolonged breastfeeding until ≥12 months was widely applied while postpartum adherence until the end of breastfeeding was poor, a potential risk of postpartum vertical transmission needs to be taken seriously into account for Option B+ implementation.

## Introduction

In 2014, about 1.2 million HIV-positive women were giving birth in the 21 priority countries for prevention of mother-to-child transmission of HIV (PMTCT) in Sub-Saharan Africa, and the number of children newly infected with HIV was still as high as 170.000.[1] Yet, it is beyond doubt that considerable progress has been achieved in PMTCT during the last five years, and UNAIDS has just announced the goal to reach less than 20.000 new infections among children by 2020. [2] The Global Plan towards the elimination of new HIV infections among children targeted a reduction of the final mother-to-child transmission (MTCT) rate to 5% or less among breastfeeding populations, and 2% or less among non-breastfeeding populations. [3] By 2014, the 21 Sub-Saharan African priority countries for PMTCT had in fact achieved an overall transmission rate of 5% after six weeks, but this increased to a final rate of 14% at the end of the breastfeeding period.^1^ Hence, breastfeeding still represents the major weakness for successful PMTCT, even though its benefit for the infant´s health in resource-poor settings is uncontroversial. [3–6]

Since 2013, WHO recommends lifelong triple antiretroviral therapy (ART) for all pregnant and breastfeeding women living with HIV regardless of their CD4-cell count and clinical stage, the so-called Option B+, as PMTCT approach wherever this is feasible to implement. [7] According to WHO, when ARVs are taken throughout the breastfeeding period as in Option B+, countries should opt to recommend breastfeeding for the first six months of life, followed by mixing suitable complementary food and continued breastfeeding from month 7–12. [8,9]

When drugs are taken as required, Option B+ allows for achieving lasting viral suppression and reducing emergence of drug resistant viral strains, and the simplified procedures and harmonization of Option B+ with general ART care are assumed to facilitate uptake and long-term adherence. [7] Yet, experiences with this approach have shown that ARV adherence and continuity of care during pregnancy and the early postpartum period are major challenges in the implementation of Option B+. [10–13] Research on later postpartum stages has been limited so far, and there is concern that ARV adherence could even more decline in the later breastfeeding period. A systematic review and meta-analysis studying perinatal adherence before the Option B+ era suggests that sufficient adherence was higher during pregnancy compared to the postpartum period [14], and barriers after delivery, such as the mother´s belief that she is cured or fear of disclosure have been described. [15,16]

Uganda faces an HIV prevalence of 7.4% [17], with a fertility rate of 5.9 births per woman [18] and an estimated number of 120.000 HIV positive pregnant women in 2013 [17]; hence, the country requires continuous engagement in effective implementation of a PMTCT approach. Uganda adopted WHO’s Option B+ strategy as one of the first sub-Saharan African countries. The roll-out began in 2012 and had reached all PMTCT facilities by March 2014. [19] After the fairly rapid nationwide introduction of Option B+, research accompanying the implementation of Option B+ in the postpartum period is rare to date, and to our knowledge, studies on feasibility during an extended breastfeeding period for up to 18 months in Uganda are entirely lacking so far. Therefore, we conducted a longitudinal study in a rural high-prevalence setting in Western Uganda to examine longer-term adherence to Option B+ and associated influencing factors until 18 months postpartum, i.e. until the end of the breastfeeding period.

## Methods

### Study setting and cohort

Within the scope of a larger PMTCT research project [11], we conducted a prospective observational follow-up study in Fort Portal, the capital of Kabarole district in Western Uganda. As of 2014, Uganda had a final MTCT rate of 8%. [1] The two major hospitals in Fort Portal, Fort Portal Regional Referral Hospital (FPRRH) and The Holy Family Virika Hospital (VH), were included in the study. Both provide standard antenatal care (ANC), post-natal care (PNC), HIV testing and treatment on-site as well as other primary healthcare and counselling services free of charge and were described in detail in a recent publication. [11]

The prospective study cohort consisted of women having been already enrolled into a larger PMTCT study at their first ANC visit [11]. Pregnant women were initially recruited and enrolled if they provided informed written consent, were above the age of 18 years, and had a positive HIV status without being on ART prior to recruitment. HIV status was determined in a routine testing sequence utilizing rapid HIV antibody test equipment (e.g., Statpack, Determine, and Unigold). ANC clients tested HIV positive between January and December 2013, were enrolled on Option B+ for PMTCT according to the national guidelines based on a single-pill fixed-dose combination of tenofovir/lamivudine/efavirenz. Drug dispensation was based on a one-month pill supply, hence requesting women to come back for drug collection monthly.

### Procedures

Participants from the larger study were enrolled into this sub-study if they returned for routine PNC at six weeks postpartum. This PNC visit at six weeks served as the first study visit for our investigation. Follow-up visits for the study were scheduled at six, twelve, and 18 months postpartum and took place until December 2015. They were aligned with routine visits for ARV drug collection, which are scheduled every four weeks in this healthcare setting. Regarding our study visits, deviation of several weeks from the scheduled date was tolerated in order to achieve a sufficiently large cohort showing three follow-up visits until the end of breastfeeding. At all four study visits, the participants were interviewed by ANC clinic staff using structured questionnaires, without interfering with routine procedures. Dried blood spots of infants were collected at all postpartum visits to determine HIV status. Standard counselling on exclusive breastfeeding for the first six months and continued complementary feeding along with breastmilk until twelve months was given in line with national recommendations. At each study visit, the appointment for the upcoming visit was scheduled. Participants not returning after the first or second study visit were defined as lost to follow up (LTFU). Data on the following socio-demographic and health care related factors had been collected at baseline in the larger PMTCT study[11] and could be examined for our sub-cohort as potential influencing factors on drug and breastfeeding adherence: age, marital status, education, occupation, obstetric history, number of members and children in the household, travel distance, travel cost, ANC attendance, and disclosure of HIV status to partner. A social status scale was established containing information on availability of electricity, tap water, radio, television, fridge, car, and a shelf in the household (scale ranging from 0–8).

Breastfeeding status and history was captured at every study visit. At the first visit, the mother´s intended feeding strategy and the infant´s drug regimen were determined. Exclusive breastfeeding (EBF) was defined as breastmilk only (plus drugs and/or vitamins as prescribed). If supplementary food, non-solid or solid, was given along with breastmilk, this was referred to as mixed feeding (MF) in infants ≤6 months, and as complementary feeding (CF) in infants >6 months. [20] Infant’s health status was determined by the study nurse and categorized as alive and well, alive with minor problems, alive with major problems, or dead.

### Measures of adherence

Drug adherence to Option B+ was assessed with two distinct measuring instruments. First, a self-rating scale for participant’s pill intake during the past month with five response categories (ranging from 1 = “took all pills” to 5 = “took no pills”) was applied. *Self reported pill intake during the last month* was assessed at every study visit. Secondly, women were asked to report the number of their drug restock visits since the last study visit. In routine PNC these were scheduled once per month. The number of restock visits was compared to the number of visits required to cover a woman´s drug supply for the particular time period. The resulting variable *difference of reported and requested drug restock visits* was created for the three intervals between the four study visits. Out of those two measures, one overall adherence category was created. A woman was defined as being fully adherent if she reported drug intake of “all” drugs during the last month, *and* if the number of her drug restock visits was in accordance with or even exceeding the required visits during the particular time span. Women not fulfilling one of these two criteria were considered as not adherent.

### Data analysis

Questionnaire data was crosschecked and entered into a Microsoft Excel database. Statistical analyses were carried out in IBM SPSS (Version 22). Descriptive statistics were performed to assess participants’ baseline information, including feeding status, duration, and infant medication. Clients LTFU were compared to returning women using t-test for continuous variables or Mann-Whitney U-test when variables were not normally distributed. Categorical data was compared using the χ^2^ -test. Associated factors to overall adherence were analyzed using non-parametric tests due to small sample size and non- normally distributed variables. Pearson´s χ^2^ or, Mann- Whitney U- test were applied for categorial or continuous data, respectively. Wilcoxon signed rank test was used to compare the two dependent variables “self-rated number of drug restock visits” and “required number of restock visits”. P-values ≤0.05 were regarded as statistically significant.

### Compliance with ethical standards

Data was used anonymously and was treated strictly confidential throughout study conduction. Informed consent was obtained from all individual participants included in the study. Women could withdraw their participation from the study at all times without explanation and without any negative consequences for their continued healthcare. The study was ethically approved by the Committee of Higher Degrees, Research and Ethics, College of Health Sciences, Makerere University, Kampala, and by the Ugandan National Council for Science and Technology.

## Results

**Fig 1:**
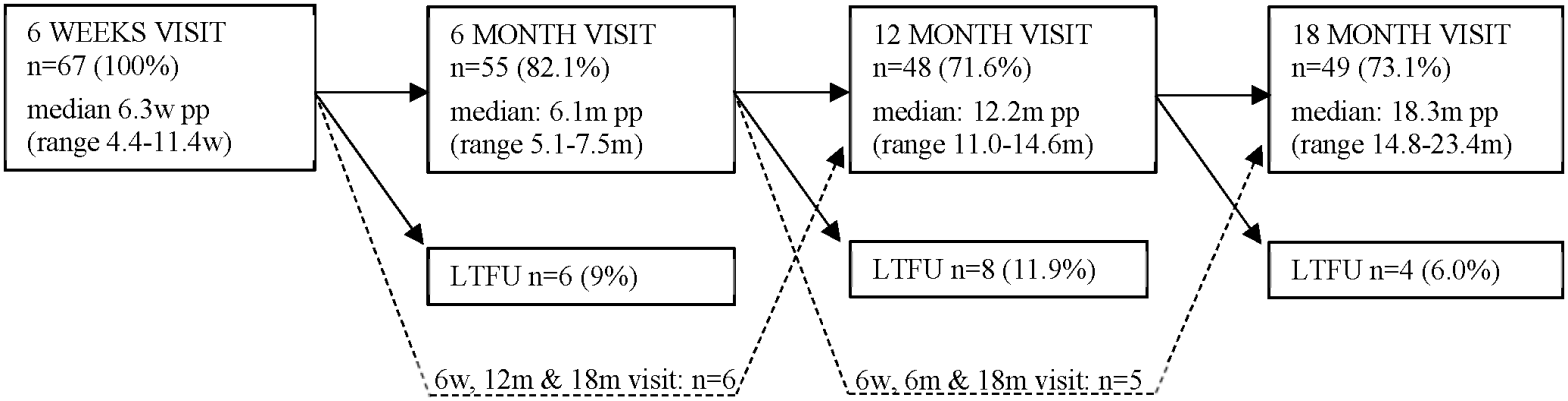
Option B+ enrolled women attending postpartum study visits.

Out of 124 HIV-positive women recruited during ANC, eight women (6.5%) were excluded due to abortion, stillbirth or early infant death. Of the remaining 116 women, 67 (58.0%) returned to the involved health facilities after delivery for their 6 weeks-visit and hence formed the cohort of this sub-study. The 67 women had given birth to 68 babies (66 singletons and one set of twins). Out of all enrolled women, 61 (91%) returned at least once in the follow-up period until 18 months postpartum, and 53 (79.1%) attended three or four visits. The number of returning clients and LTFU per visit are displayed in Fig 1.

### Baseline characteristics

Sociodemographic and clinical characteristics of the study cohort are shown in Table 1. Fourteen women (20.9%) were LTFU within the first six months. Comparing them to participants retained in care for twelve months or longer (n = 53; 79.1%) revealed a significant age difference with women LTFU being significantly younger (mean 22.1 years) than followed-up clients (mean 26.7 years; p = 0.006). Among clients LTFU, 9/14 (64.3%) had disclosed their HIV status to their spouse whereas among the followed-up clients 47/52 (90.4%) had done so (p = 0.029).

**Table 1:** Sociodemographic and clinical characteristics of the study cohort.

Variable	
**Enrolled postpartum clients**	67
VH	38 (56.7%)
FPRRH	29 (43.3%)
**Number of antenatal care visits (n = 67)**	
Median (range)	4.0 (1–7)
**Number of postpartum study visits (n = 67)**	
Median (range)	3.0 (1–4)
**Age (n = 67)**	
Median (range)	25.0 (18–39)
**Educational degree (n = 67)**	
None or primary education only	39 (58.2%)
Higher than primary	28 (41.8%)
**Marital status (n = 67)**	
Married	44 (65.7%)
Single/unmarried/widowed/divorced	23 (34.4%)
**Occupation (n = 63)**	
No income-generating activity	31 (49.2%)
Income-generating activity	32 (49.8%)
**Social Status Index (0–8, n = 67)**	
median (range)	3.0 (0–8)
**Number of household members**^a^ **(n = 61)**	
median (range)	3.0 (1–10)
**Number of children in the household**^a^ **(n = 59)**	
median (range)	1.0 (0–7)
**Travel distance (minutes, n = 59)**	
median (range)	30.0 (0–180)
**Travel cost (UGX, n = 62)**	
median (range)	2000 (0–10000)
**Previous deliveries**^a^ **(n = 65)**	
median (range)	1.0 (0–7)
**Delivery mode (n = 67)**	
Spontaneous delivery	61 (91.0%)
Cesarean section	6 (9%)

^a^ data collected antepartum

### Maternal drug adherence

According to the study´s definition of full adherence, three participants (4.5% of all) were adherent until 12 months i.e. during the 6 weeks-6 months interval and during the following 6 months-12 months interval. However, no single client was fulfilling the criteria during the entire follow-up period until 18 months. Analyzing overall adherence and associated factors for every follow-up visit separately, our definition of full adherence applied to 26/51 participants (51.0%) at the 6 weeks-6 months interval, to 8/42 participants (19.0%) at the 6–12 months interval, and to 9/44 participants (20.5%) at the 12–18 months interval (Table 2).

**Table 2:** Maternal adherence at different study visits.

Maternal Adherence Variables		6 months postpartum	12 months postpartum	18 months postpartum
**Self-reported complete pill intake during last month**	n (%)	53 (96.4%)	48 (100.0%)	47 (95.9%)
**Difference of reported and requested drug restock visits**^a^	median(range)	0 (-5–3)	-2 (-5–5)	-2 (-9–12)^b^
**Overall adherence category**^c^				
Adherent	n (%)	25 (49.0%)	8 (19.0%)	9 (20.5%)
Non-adherent	n (%)	26 (51.0%)	34 (81.0%)	35 (79.5%)

^a^ Number of reported drug restock visits compared to the number of visits required to cover the woman´s drug supply for the particular time interval, with negative values indicating missing drug restock visits for the time interval

^b^ More than 6 requested visits were noted for women where follow-up time intervals deviated from the 6-months interval, according to the length of their interval.

^c^ Created out of the first two variables, i.e. self-reported pill intake and sufficient drug restock visits

We also analysed the two scales separately that determined our “full adherence” category. Self-rated adherence was high throughout all four study intervals with ≥95% of clients claiming that they had taken all pills. However, drug restock visits, which were part of the PNC routine, were performed less frequently than required: There was no difference between reported drug collection visits and required amount of visits at six months postpartum, but a median of two restock visits was missing for the preceeding time periods at twelve and 18 months postpartum (Table 2). Throughout all study visits, clients median number of drug collections was 11.0 compared to a median of 15.0 required drug collection visits (p = 0.01).

Mothers non-adherent at six months postpartum had a higher median number of previous deliveries (p = 0.015) and higher travel costs to hospital (p = 0.024). Longer intended duration of complementary breastfeeding was associated with full adherence (p = 0.035). However, there was no significant link between actual breastfeeding duration and postpartum adherence. Comparing adherent and non-adherent mothers one year after delivery revealed significantly older age (p = 0.001) and higher number of previous deliveries (p = 0.039) in non-adherent mothers (Table 3). At eighteen months postpartum, none of these differences were found to be significant between the adherent and the non-adherent participants.

**Table 3:** Sociodemographic and clinical characteristics by adherence category.

Variables	n	Adherent 12 months Postpartum	Non-adherent 12 months postpartum	p-value^a^
	42	(n = 8)	(n = 34)	
**Age at first ANC visit**				
*Median (range)*		20 (20–25)	28 (18–38)	**.001**
**Educational degree** *n (%)*	**42**			
None or primary	25	6 (24.0)	19 (76.0)	.282
Higher than primary	17	2 (11.8)	15 (88.2)	
**Marital status** *n (%)*	**42**			
Married	25	3 (12.0)	22 (88.0)	.156
Single/widowed/divorced	17	5 (29.4)	12 (70.6)	
**Occupation** *n (%)*	**40**			
No income-generating activity	23	4 (17.4)	19 (82.6)	.489
Income-generating activity	17	2 (11.8)	15 (88.2)	
**Disclosure of HIV status** *n (%)*	**41**			
Status disclosed	38	8 (21.1)	30 (78.9)	.512
Status not disclosed	3	0 (0)	3 (100)	
**Social Status Index**	**42**			
*median (range)*		2.5 (1–8)	3 (1–8)	.210
**Travel distance** (in minutes)	**34**			
*median (range)*		20 (10–60)	30.0 (0–180)	.741
**Travel costs** (in UGX)	**40**			
*median (range)*		3000 (1000–7000)	2000 (0–10000)	.263
**Number of household members**	**38**			
*median (range)*		2.5 (1–7)	3.5 (1–10)	.368
**Number of children in the househould**	**38**			
*median (range)*		0 (0–3)	2 (0–7)	.050
**Previous deliveries**	**40**			
*median (range)*		0.5 (0–2)	2 (0–7)	**.039**

^a^ Pearsons Chi^2^ or Mann-Whitney U-Test

### Breastfeeding duration and infant health

Most participants (91%, n = 61) were lactating exclusively for the first six months, while five women did so for four months or less (1 missing data). The median breastfeeding duration was 12.0 months (range 2–18 months). The WHO- recommended breastfeeding period of at least 12 months was realized by 76.5% of the participants followed-up (12 months, n = 38; 18 month, n = 1), while at the first visit, 55/67 women (83.6% of all) had expressed their intention to do so. A shorter breastfeeding period of 10 month was reported by two (3.9%) of the followed-up women. Two participants failed to give retrospective information on feeding duration.

ARV coverage for infants (nevirapine for six weeks postpartum) exceeded 90%. At the first study visit, 62 infants (91.2%) had been receiving ARVs since delivery. Among those six infants who had not received ARV prophylaxis immediately after delivery, four had received Nevirapine until the second study visit, and two were LTFU. At all four study visits, ≥95% of infants were considered as being well, and no child had major health issues at any visit. All infants were HIV negative during their first PCR at six weeks, and remained seronegative throughout the entire breastfeeding period. Until 18 months postpartum, there was no case of HIV transmission observed in the study cohort.

## Discussion

Our longitudinal study in rural Uganda is among the first to observe adherence to Option B+, breastfeeding duration, and MTCT rates from delivery until 18 months postpartum. Overall, we found that despite the relative convenience of simplified procedures in Option B+, no mother from our cohort managed to fully adhere to it until 18 months postpartum. At the six-month postpartum visit, half of all clients fulfilled our definition of adherence, declining to only three clients fully adherent throughout 12 months.

Our finding of suboptimal postpartum adherence coincides with other studies based in sub-Saharan countries. In a recent study conducted in Zambia, Okawa et al. found self-reported adherence to Option B+ in 80% of clients from pregnancy to six weeks postpartum, and a decrease to 70% at 24 weeks postpartum. [13] This is comparable to our finding of high self-reported adherence. Moreover, among previous studies focusing on postpartum Option B+ adherence, follow-up periods are shorter and adherence is often defined as retention in care. Studies conducted before the Option B+ era found comparably low postpartum adherence to PMTCT care. In rural Tanzania, adherence in terms of correct dispensation of drugs after delivery was seen in 78% of participating mother-infant pairs. [21] In a cross-sectional study conducted in South Africa, 86% of participating women who were asked about their postnatal adherence to regular azidothymidine intake reported that they were adherent the last four days prior to the interview. [22] Another study from Uganda found a 38% adherence rate for meeting a scheduled appointment six-weeks postpartum. [23] The design, adherence definition and recommended drug regimens in these studies differed from the conditions in our study, thus, respective adherence levels are not comparable. This represents a general problem in adherence-related research, and further investigations targeting the comparability and also the reliability of various adherence measures would be extremely helpful.

Research on Option B+ adherence during pregnancy usually found better adherence. In an Ethiopian study, 87.1% of participants were reported to be adherent in self-reported drug intake. [10] A Kenyan study revealed predelivery adherence rates of 84% [24], and in Uganda, a median pill count adherence level of 95% was found throughout pregnancy. [11] As a matter of a fact, the relatively high adherence to Option B+ in ANC seems to decline in the postpartum period, particularly in the later breastfeeding stage, as observed in our cohort. We found that from the clients having been enrolled in the larger PMTCT study, only 58% returned after delivery for postnatal care. As our study visits were scheduled in line with routine PNC/drug refill services, LTFU corresponds to poor retention in care in the postpartum period. Future research should focus on how to retain women in PMTCT services at this point of the PMTCT cascade.

Our findings also raise the question how barriers for adherence to Option B+ might be different in the postpartum period compared to the antenatal period. [16] A factor previously suggested, and presumably specific to the postnatal period, is the belief that HIV care for the mother´s own health is irrelevant once the infant is born, especially after a baby is proven to be HIV-negative. [15] Moreover, mothers who do not suffer from clinical symptoms might not feel a strong need to adhere to health care appointments and daily drug intake. Previous research suggests that mothers who initiated ART for PMTCT are more likely to drop out of care after delivery than women starting ART for their own health [12, 25].

According to our findings, adherence was higher in the first six pospartal months compared to later observation periods. This implies that particularly the later part of the breastfeeding period should be in the focus of attention of health care providers regarding adherence motivation to avoid late stage transmission. Phone calls or SMS text messaging have proven to be effective in increasing retention in several studies. [26–28] Furthermore, assignment of community health workers [29], as well as adherence counselling training for health care staff [30] were previously used to increase adherence in pregnancy and early infant diagnosis; these strategies should be adapted to the breastfeeding period. For health service implementers, it is highly relevant to understand in which time interval after delivery it could be especially difficult for mothers to adhere to drug regimens or pick up their drugs from the health facility. This knowledge could lead to specific support activities for specific time intervals e.g., repeated adherence counselling in the later postpartum stage, rendered to Option B+ clients by the health facilities.

Socioeconomic determinants to adherence and retention during the entire PMTCT cascade have been summarized in previous reviews. [16,31,32] We found that postpartum non-adherence until six months after delivery was associated with higher travel costs. Previous research similarly identified structural barriers such as long travel distance to the health facility, often linked with higher travel expenses. [33,34] Another significant influencing factor was found in higher number of previous deliveries, serving as a proxy for the number of children a woman already has at home, hence for the burden of workload she is facing regarding child care. To overcome structural barriers like distance, time and travel funds, health policy makers and governmental institutions should take into consideration how these could be targeted on a lasting basis. Home-based care [35] for those who cannot leave their homes due to child care duties or travel costs might also be an option worthwhile debating.

In our study, three quarters of our participants adhered to the recommended 12-month breastfeeding period with six months of exclusive breastfeeding, demonstrating a broad acceptance of the paradigm shift from abrupt weaning after six months towards a recommended 12-month breastfeeding period for women living with HIV. This is in accordance with findings from Ngoma et al., in which mothers reported a high adherence to breastfeeding recommendations. [36] Mothers’ strong commitment to having a healthy, well-nourished baby [37] could explain the high compliance with breastfeeding recommendations, and represents a factor that interventions for increased PMTCT adherence should focus on. However, continued breastfeeding for 12 months as recommended by WHO is strongly and inevitably linked to the precondition that the mother is under ART/Option B+. [7,8] Without sufficient viral suppression, prolonged breastfeeding could lead to high postnatal vertical transmission rates [38], as in the early times of short-term PMTCT regimens. Our finding that in the Option B+ era, HIV-positive mothers widely apply the recommendation for longer breastfeeding on the one hand, but on the other hand do not adhere to continuous drug intake throughout infant exposure to breastmilk is therefore rather alarming. Health authorities urgently need to focus on strengthening postpartum adherence when deciding that health services should counsel in favor of prolonged breastfeeding, in order to avoid a setback in MTCT rates.

We found no case of vertical transmission in our study cohort. While possibly pointing to a high effectiveness of Option B+ despite suboptimal adherence, such conclusions should be drawn with caution in the light of our relatively small sample size. Apart from that, it is possible that HIV-infected infants were among the cases LTFU, possibly having deceased within the first 18 months of life, and results from our followed-up group could be underestimating true transmission rates. For conclusive appraisals on HIV transmission after 18 months under Option B+, larger cohorts are urgently needed, and following-up the dropout cases would be highly elucidating.

The rather small sample size was also a limitation for our study in terms of not permitting multivariate analysis, which would have strengthened our assessment of factors influencing adherence. Yet, considering the immense scale-up of HIV testing and ART programs in the past years, it has become challenging to obtain large PMTCT cohorts, and related research will have to focus on multicenter studies in order to achieve high patient volumes [11]. At the same time, longitudinal cohorts over a period of 18 months are prone to LTFU, further decreasing the final sample size. The problem of high drop-out rates during follow-up are a common limitation in PMTCT-related research in general [34,39,40]. We tried to mitigate this problem by aligning study visits with routine healthcare visits; yet, dropout rates in the course of the postpartum period were high, and actively following-up on these dropouts was beyond the scope of our study. However, given the scarcity of respective longitudinal studies, we believe that we can still provide meaningful insights in terms of adherence to Option B+ in the postpartum period.

Another limitation of this research is found in the fact that self-reported adherence is prone to social desirability and recall bias and hence to an overestimation of adherence. In our study, we aimed at mitigating those biases by not only relying on self-reported adherence, but combining it with measuring sufficiency of drug restock visits, as well as by creating a nonjudgemental interview setting within the study to facilitate honest reporting. Beyond that, it would give helpful insight on adherence measurement to triangulate self-reported adherence with pill count and plasma drug levels in future research. Finally, we acknowledge that adherence to drug dispensary visits does not necessarily equal drug intake, again underlining the potential risk of overestimating adherence. Still, if true, this only would reinforce our finding of suboptimal adherence.

In conclusion, we identified long-term drug adherence to Option B+ until 18 months postpartum to be suboptimal, pointing at enduring challenges in the implementation of this strategy. Meanwhile, the WHO recommendation for continuous breastfeeding until 12 months and beyond was widely applied by HIV-positive women under Option B+. Low drug adherence clearly compromises the effectiveness of Option B+, and it should be stressed that especially women who prolong the breastfeeding duration for their baby while at the same time not adhering to ARV intake put their infant at high risk for infection. Our findings emphasize a need for postpartum interventions encouraging drug adherence among women taking Option B+, especially in later stages of breastfeeding.
